# The Diverse Contributions of Fucose Linkages in Cancer

**DOI:** 10.3390/cancers11091241

**Published:** 2019-08-24

**Authors:** Tyler S. Keeley, Shengyu Yang, Eric Lau

**Affiliations:** 1 Department of Cellular and Molecular Physiology, Penn State College of Medicine, Hershey, PA 17033, USA; 2 University of South Florida Cancer Biology Graduate Program, Tampa, FL 33602, USA; 3 Department of Tumor Biology, H. Lee Moffitt Cancer Center, Tampa, FL 33602, USA

**Keywords:** fucose, fucosylation, fucosyltransferase, cancer, signal transduction

## Abstract

Fucosylation is a post-translational modification of glycans, proteins, and lipids that is responsible for many biological processes. Fucose conjugation via α(1,2), α(1,3), α(1,4), α(1,6), and O’- linkages to glycans, and variations in fucosylation linkages, has important implications for cancer biology. This review focuses on the roles that fucosylation plays in cancer, specifically through modulation of cell surface proteins and signaling pathways. How L-fucose and serum fucosylation patterns might be used for future clinical diagnostic, prognostic, and therapeutic approaches will be discussed.

## 1. Introduction

Fucose is a natural deoxyhexose sugar with similar structure to glucose, except for its lack of a hydroxyl group on carbon 6. Mammalian cells utilize fucose in the L-enantiomer form, whereas other deoxyhexoses are used in the D-enantiomer. L-Fucose is incorporated onto glycoproteins during the synthesis of N- and O-linked glycans in mammalian cells [1,2]. Fucosylated glycans elicit a range of functions from regulating inflammatory responses, signal transduction, cell growth, transcription, and adhesion [3]. For example, cell-cell interactions can be partially modulated by the presence of L-fucose specific-lectin-like adhesion molecules on the cell surface [4]. In addition, the fucosylation of cell membrane receptors and proteins, including EGFR, TGFβ, Notch, E-cadherin, integrins, and selectin ligands, has been reported to influence their ligand binding, dimerization, and signaling capacities [5,6,7,8].

Cancer is characterized by the deregulation of otherwise normal cellular and molecular processes, which can restrict or suppress tumorigenesis, resulting in altered growth, survival, metabolism, and metastasis [9]. Post-transitional modifications, including fucosylation, represent an important regulatory layer that is subject to perturbation during carcinogenesis and tumor progression. Increasing numbers of studies have identified important and specific roles for fucosylated glycoconjugates in tumorigenesis and how they correlate with the established hallmarks of cancer [10]. Deregulation of fucosylation has been reported in several cancer types, and the resulting divergent functional consequences are likely attributed to the presence/absence of specific structural types of fucosylation branching that can differentially influence protein function [11,12]. Increased fucosylation has been attributed to metastatic properties such as, for example, enhancing adhesion of metastatic cancer cells to endothelia cells [1,4,13]. However, fucosylation has also been reported to suppress motility in cancers such as melanoma [14,15], oral/head and neck [16], and hepatocellular carcinoma (HCC) [17]. Here, we have examined and summarized the literature in order to highlight alterations in fucosylation across various cancer types and whether fucosylation branches are associated with divergent pathological phenotypes.

## 2. Fucose Metabolism

**Figure 1:**
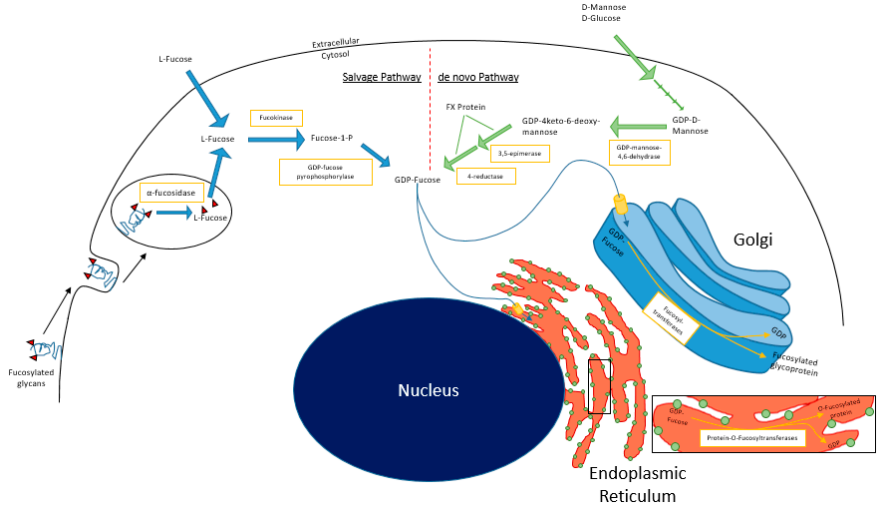
Fucose salvage and de novo pathways.

In mammalian cells, fucosylation starts with substrate (GDP-fucose) generation, which can occur via the *de novo* and/or salvage pathways (Figure 1). In the *de novo* pathway, GDP-mannose in the cytosol is converted to GDP-4-keto-deoxymannose by GDP-mannose 4,6-dehydratase (GMD). The keto intermediate is rapidly converted to GDP-4-keto-6-deoxygalactose by the NADP(H)-binding epimerase-reductase FX protein to GDP-fucose [1]. In the salvage pathway, L-fucose is transported into the cytosol from the extracellular space or from lysosomal compartments, by mechanisms that remain relatively undefined in mammalian cells [18]. Free L-fucose in the cytosol is phosphorylated by fucokinase (FUK). The resulting fucose-1-phosphate is converted to GDP-fucose by GDP-pyrophosphorylase [1]. GDP-fucose is then transported into the Golgi apparatus, where it is used as a substrate for protein fucosylation that is mediated by fucosyltransferases. Alternatively, GDP-fucose can also be conjugated onto proteins by Protein O-fucosyltransferases (POFUTs) in the endoplasmic reticulum [19,20,21]. A recent study by Ng et al. reported that human patients with pathogenic mutations in FUK present with severe developmental impairments including encephalopathy, hypotonia, and seizures [22]. These data suggest tissue type-dependent reliance on *de novo* synthesis vs. salvage pathway-derived GDP-fucose.

The main source of L-fucose for the salvage pathway comes from diet, predominantly from plant sources such as seaweed [23]. However, when dietary sources are insufficient, L-fucose can be catabolized from preexisting fucosylated glycoconjugates or supplemented by the *de novo* pathway. The glycoconjugates are endocytosed and catabolized in lysosomes, where fucosidases liberate L-fucose from the glycans, rendering them once again useable via the salvage pathway. This mechanism has been reported to generate sufficient GDP-fucose to sustain physiological functions of the cell when *de novo* pathway activity is insufficient or pathologically abrogated, provided that exogenous supraphysiological L-fucose concentrations are available to cells [1,24].

## 3. Fucosyltransferases: Architects of Fucosylation Branching

**Figure 2:**
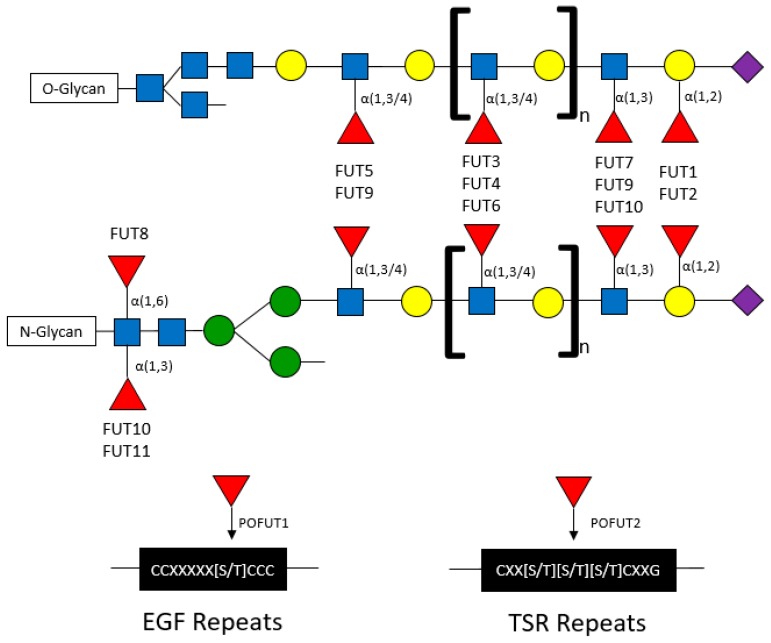
Fucosyltransferases and their associated conjugated fucose structures.

Fucose can be conjugated onto oligosaccharides in the following specific orientations: α(1,2), α(1,3), α(1,4), and α(1,6) orientations, where the first carbon of fucose is bound to the (1,2), (1,3), (1,4), or (1,6) carbon of galactose or N-acetylglucosamine (GlcNAc) [25,26]. The α-fucose conjugations can occur at core or terminal positions along glycans (Figure 2). Core fucosylation has been reported to play important roles in influencing the signaling capacity of membrane-bound proteins including EGFR. T cell receptors are heavily core fucosylated, which ensures proper activation and downstream signaling [27]. Notably, only FUT8 has been identified to mediate core fucosylation (*via* “N’-linkages”), wherein fucose is conjugated to a primary N-acetylglucosamine branch (GlcNAc) on N-glycans [28]. Terminal fucosylation refers to fucose conjugated to GlcNAc monosaccharides (also known as α(1,3) or α(1,4) branching) or to galactose residues (also known as α(1,2) branching) (Figure 2) [2,28].

Terminal fucosylated structures are highly diverse, contributing to the generation of Lewis antigens on a multitude of proteins [29]. The topological landscape of fucosylated glycans and their functional consequences are dictated by fucosyltransferases (FUTs). Methods for detecting specific fucosylated structures include mass spectrometry, as well as binding/pulldown approaches using lectins with binding affinities for fucosylated structures (Table 1).

**Figure 3:**
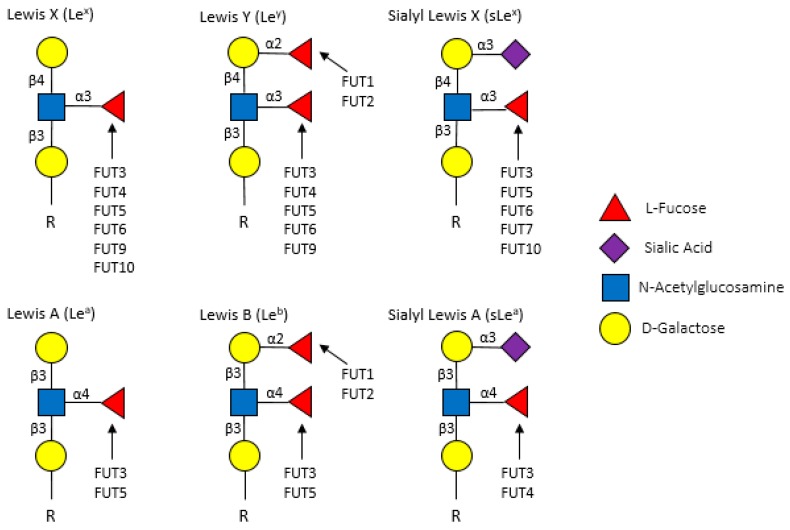
Lewis antigens commonly found in on the surface of cancer cells.

Fucosyltransferases are membrane-bound proteins residing in the endoplasmic reticulum and Golgi. To date, 11 FUTs and 2 protein O-fucosyltransferases (POFUTs) have been discovered. FUTs and POFUTs transfer fucose using GDP-fucose as a substrate to oligosaccharides, glycans, lipids, and proteins to form fucosylated glycoconjugates [21,28,30,31]. Additionally, fucose can be directly O-link conjugated onto serine or threonine residues of Epidermal Growth Factor (EGF)-like repeats by POFUT1 and Thrombospondin Type 1 (TSR) repeats by POFUT2 [19,20,21,28]. The vast array of FUT-mediated fucosylation (and fucosylated target proteins) has been extensively characterized in non-cancer, particularly immunological, contexts. However, many of the immune proteins known to be fucosylated might also play roles in cancer. For example, the expression of Lewis (Le) antigens, which are oligosaccharide-based blood antigens containing differing orientations of fucosylation (Figure 3), correlate with cancer stage, tumor cell differentiation, decreased survival, and metastasis [32].

Fucosyltransferases can generally be grouped by the fucosylation linkages that they mediate (Table 2). FUT1 and FUT2 mediate α(1,2) fucosylation on terminal galactose residues on both O- and N-linked glycans. FUT3-7 and FUT9-11 are responsible for the addition of fucose to GlcNAc monosaccharides in α(1,3) and α(1,4) orientations on O- and N-glycans. FUT8 is the only transferase that has been shown to conjugate fucose to the initial GlcNAc residue on N-glycans in α(1,6) branching (core fucosylation) structures. Although extensive studies have investigated the structural fucosylation linkages and functional effects mediated by these FUTs, the fucosylated targets are largely not well characterized.

## 4. Serum Fucose and Fucosylated Glycoconjugates

Suboptimal diagnostic modalities represent an ongoing clinical challenge, hampering timely and efficient detection and treatment of cancer until it has progressed to advanced stages. The development of innovative early detection approaches is crucial for improving survival probability and the quality of life for cancer patients. Recently, the profiling of cancer patient sera for altered glycosylation states/levels of secreted proteins has emerged as a promising new diagnostic approach. Methods of detection have included high performance liquid chromatography (HPLC), liquid chromatography (LC), mass spectrometry (MS), matrix assisted laser desorption/ionization (MALDI), or combinations therein. In Table 3, we summarize findings from studies comparing serum fucose and fucosylated glycan profiles from healthy individuals vs. breast, oral/head and neck, HCC, ovarian, CRC, pancreatic, lung and prostate cancer patients. Several of these studies suggest diagnostic potential for serum L-fucose levels, which have been found to be elevated in cancer patient sera in breast [53,54,55], oral/head and neck [56,57,58,59,60,61], HCC [62,63,64,65], CRC [66,67], and ovarian [55,68] cancer patients compared with healthy individuals. Specific fucosylated glycoconjugates have been found to also have potential diagnostic utility. For example, fucosylated haptoglobin correlates with poorer survival probability, reduced responsiveness to therapy, and increased metastatic burden in breast [31], HCC [69,70,71], ovarian [31,55,72], CRC [67], pancreatic [73], and lung [31,74] cancer patients. Similar to haptoglobin, levels of serum fucosylated α-fetoprotein are also increased in HCC patients have been reported to correlate with poor survival outcomes, increased disease recurrence, and portal vein thrombosis [31,75].

Importantly, specific cancer types, such as lung cancer, can be further segregated into subgroups by fucosylation status. For example, a MALDI-MS comparison of the sera from former and current smokers with non-small cell lung carcinoma (NSCLC) revealed that fucosylated glycans were increased in current smokers [84]. Notably, serum fucose/FUT activity has been reported to associate with prognosis/therapeutic responsiveness in some cancers. Breast cancer and CRC patients undergoing chemotherapy or surgical resection, respectively, exhibit reduced serum FUT activity after therapy, suggesting (i) that serum FUT activity predominantly originates from the tumors, and (ii), that serum FUT activity/fucosylation levels might reflect therapeutic responsiveness [4,76]. Consistent with this notion, serum levels of α(1,3) fucosylation in breast and ovarian cancer patients are elevated during tumor progression but are significantly reduced in patients who responded to chemotherapy [55].

Despite growing evidence that aberrant fucosylation correlates with staging in several cancer types, the nature of correlative vs. causative relationship between differences fucose linkages on serum glycoconjugates and cancer is poorly understood. For example, whereas the α(1,2)-fucosylated serum species of prostate specific antigen (PSA) has been reported to exhibit stronger correlation with the presence of prostate cancer than total PSA, the α(1,6)-fucosylated species correlates with metastasis [81,82,83,85]. Increased serum α(1,3)-fucosylated sLe^x^ antigen or α(1,6)-fucosylated haptoglobin levels are associated with the presence of breast cancer or HCC, respectively [54,70]. Similarly, core fucosylated α-fetoprotein has exhibited clinical utility in the early detection of HCC [3]. Interestingly, increased serum levels of fucosylated E-cadherin also correlates with stage-independent poor prognosis in lung cancer patients [51]. The diversity of linkage types and the largely unknown/uncharacterized fucosylated proteins and their functional contributions to cancer represent an area of opportunity for important and clinically relevant basic research.

## 5. α(1,2) Fucosylation

α(1,2) fucosylation exhibits seemingly divergent effects in cancer progression. Whereas it is tumor suppressive and reduced in melanoma [14,15,86,87,88], oral/head and neck [16], gastric [89], and HCC [17,63] carcinomas, it is tumor-promoting and increased in bladder, breast, epidermoid, ovarian, and prostate tumors (Table 4) [33,90,91,92,93].

To date, α(1,2) fucosylation levels in cancer correlate with, and appear most likely regulated by, FUT1. The downregulation of FUT1, and consequently of α(1,2) fucosylation, has been attributed, at least in pancreatic cancer, to constitutive Hif1α-mediated transcriptional suppression, suggesting that in pancreatic cancer, hypoxia suppresses cell surface α(1,2) fucosylation, which promotes cancer cell motility and migration [101]. We recently reported that FUT1-mediated α(1,2) fucosylation abrogates invadopodia formation/ECM degradation and inhibits melanoma cell motility and tumor growth/metastasis in vivo [14,87,88]. These findings indicate that reduced FUT1 expression, and consequently, loss of α(1,2) fucosylation, promotes melanoma invasiveness/progression by enhancing invadopodia formation. The cell-based studies and in vivo xenograft models are consistent with findings from OSCC and HCC cells, supporting the roles of FUT1 and α(1,2) fucosylation in suppressing tumor progression and metastasis.

In contrast to melanoma, OSCC, and HCC, α(1,2) fucosylation is increased and elicits tumor-promoting effects in bladder, breast, epidermoid, ovarian, and prostate tumors, where it stimulates cellular proliferation, adhesion, invasion, metastasis, and drug resistance [11,33,91,93,103,104,105,106]. In ovarian [96] and prostate [85] tumors, increased α(1,2) fucosylation is linked to upregulation of FUT1 and promotes TGFβ signaling, cellular proliferation, and impairs apoptosis [36,96,107]. The precise molecular mechanisms remain unclear and likely require functional players beyond TGFβRI/II. For example, in ovarian cancer, FUT1 promotes upregulation of MUC1, which stimulates proliferation, invasion, and metastasis [108]. In breast cancer, FUT1 promotes mTOR activity and lysosomal and autophagosomal dynamics via α(1,2) fucosylation of lysosome-associated membrane protein (LAMP) 1 [91].

However, α(1,2) fucosylation/FUT1 can also elicit seemingly divergent tumor-suppressive or tumor-promoting effects in cancer. For example, the ectopic expression of FUT1 in CRC cells perturbs their stromal interactions in vitro and impairs metastatic capacity in vivo [17,97,109]. Although α(1,2) fucosylation thus appears to be tumor-promoting in CRC, it has also been reported to be upregulated in patient-derived CRC tissues compared to normal tissues [97,98,99]. This dichotomy might be due tumor stage-specific functions/effects of FUT1, where loss of α(1,2) fucosylation is required before metastatic cells can adhere to new sites. The expression of FUT1 is decreased in pancreatic primary tumor cell lines compared to normal tissue, but the ectopic re-expression of FUT1 in the metastatic pancreatic cancer cells inhibits metastasis by enhancing the cell surface abundance of Le^y^ and inhibiting E-selectin-mediated adhesion [17,86]. Together, these studies suggest that α(1,2) fucosylation plays crucial tumor suppressive roles during initiation and that its loss promotes metastatic progression.

Given the remarkable heterogeneity between cancer types, further studies will be crucial for elucidating the specific fucosylated proteins (e.g., key upstream receptors, stromal-interacting membrane proteins etc.) that mediate the divergent effects elicited by α(1,2) fucosylation to promote/suppress tumor progression. Furthermore, the identification of cancer signaling pathways that are significantly altered by α(1,2) fucosylation, together with the identification of key fucosylated proteins mediators, might yield useful insights for the stratification/therapeutic intervention for subsets of cancer.

## 6. α(1,3) and α(1,4) Branching

Compared to α(1,2) fucosylation, more consistent pathological effects have generally been reported for α(1,3/4) fucosylation across different cancer types. α(1,3/4) fucosylation is upregulated in breast [110,111,112], liver [63,113], ovarian [114,115], CRC [97], pancreatic [100,116,117], gastric [118,119], lung [120], and prostate [121,122,123] cancers compared with normal tissue counterparts (Table 4). Several of the above-mentioned studies focused on a single or a few FUTs using RT-PCR, Le antigen IHC staining, ELISAs with corresponding lectins, and/or lectin microarrays and have reported that increased levels α(1,3/4) fucosylation contribute to metastasis. As detailed in Table 5, several FUTs can mediate α(1,3/4) fucosylation that confers tumorigenic properties.

Of the FUTs that mediate α(1,3)- and/or α(1,4)-fucosylation, FUT3, 4, 6, and 7 are most frequently reported as upregulated across cancer types. Of the other FUTs, FUT5, FUT10, and FUT11 have been reported to contribute to cell adhesion and metastasis through the generation of sLe^x^ and sLe^a^ antigens [111,119]. In breast cancer cells, increased FUT4, 5, 6, 10 and 11 levels correlate with increased migration and proliferation and the increased expression of angiogenesis-related genes including VEGFA, VEGFR1, VEGFR2, and FGF2. Pharmacological inhibition of fucosylation using 2-fluorofucose, a fucosyltransferase inhibitor, blocks breast cancer cell migration and proliferation and is associated with attenuated RTK, MAPK and p38 signaling [111]. In ovarian cancer cells, FUT3, 4, and 9 promote motility by mediating the α(1,3) and α(1,4) fucosylation of specific Le antigens [115,126].

FUT3 has generally been reported as a crucial mediator of tumor-promoting signaling. In CRC, FUT3 is required for TGFβ signaling, as knockdown of FUT3 inhibits fucosylation of TGFβR1 and attenuates Smad2 signaling, consequently decreasing migration and invasion [127]. Loss of FUT3 across several tumor cell lines/types has been reported to decrease migration [119,127], invasion [127], TGFβ signaling [127], interaction with E-selectin [116,119,123,125], metastatic potential in vivo [116], and drug resistance [128]. In contrast, the ectopic overexpression of FUT3 amplifies sLe^x^ levels [122,124] and promotes cellular adhesion [122], tumor growth [122], and metastasis [122,124].

FUT4 is upregulated in several cancer types and has been shown to promote proliferation [129], invasion [125,130], tumor growth [129,130], and drug resistance [128,131]. Consistent with pro-tumorigenic function, loss of FUT4 in melanoma cells inhibits proliferation and tumor growth and is associated with reduced EGFR and MAPK signaling [129]. FUT4 is also implicated in drug resistance. For example, the ectopic expression of FUT4 in multidrug-resistant HCC cells enhances activation of pro-survival signaling including the PI3K/AKT pathway [131]. However, FUT4 has been reported to elicit anti-tumor effects. For example, the ectopic expression of FUT4 in A549 lung cancer cells suppresses EGFR signaling and invasive capacity [6]. It is possible that opposing tumorigenic vs. tumor-suppressive functions of FUT4 are elicited in stage- and context-specific manners. Recently, FUT4 expression was shown to be regulated by several miRNAs, which are downregulated in breast cancer tissues [92,130,132], highlighting one mechanism by which FUT4 fucosylation is enhanced in breast cancer tissues. It is possible that other FUTs are subject to similar mechanisms of regulation.

FUT6 has also been reported to elicit similar pro-tumorigenic roles as FUT4 in various cancer types [113,125,127,131,133]. Like FUT3, FUT6 also fucosylates sLe^x^ antigens, amplifying cellular adhesion and promoting metastasis, with concomitant upregulation of pro-tumorigenic TGFβ signaling [127].

FUT7 is upregulated in HCC [134,135,136], lung, [137] and prostate [133] carcinomas and elicits tumor-promoting effects. The ectopic expression of FUT7 promotes adhesion, colony formation, invasion, proliferation and survival [108], and migration [133,134,138], whereas its knockdown reverts these effects. Although the effects appear to require p38 and JNK, the direct underlying mechanisms are currently not known [136].

In summary, α(1,3/4) fucosylation, which is mediated by FUTs 3-7 and 9-11, is generally increased and elicits tumor-promoting effects in the cancers discussed above. This has been evidenced by the fact that these FUTs mediate the production of several Lewis antigens, including sLe^x^, which have been demonstrated to promote metastatic capacity. Specifically, sLe^x^, which is upregulated in cancer cells, can promote metastasis by binding to E-selectin, which is expressed on endothelial cells. This interaction can slow the rolling speed of cancer cells along the vascular endothelium under shear forces, enhancing the ability of circulating tumor cells to extravasate from the vasculature into surrounding tissues [116,119,121,123,134]. In addition to increasing sLe^x^ levels, several α(1,3/4) FUTs can alter cell surface receptor (e.g., growth factor receptor)-mediated signaling, which is important for tumor development. Future comprehensive studies are required to dissect the probable complex functional redundancy among the α(1,3/4) FUTs to determine their specific cancer type- and stage-specific functional contributions. Ultimately, the elucidation of the pathological contributions of α(1,3/4) FUTs is important for developing novel therapeutic targets and strategies.

## 7. α(1,6) Fucosylation

FUT8 is the only fucosyltransferase known, to date, to conjugate fucose onto core GlcNAc residues of N-glycans. Extensive studies in melanoma [95], breast [139], liver [63,95,140], ovarian [68], cervical [141] CRC [97,142,143], pancreatic [117], gastrointestinal [89,144,145,146], thyroid papillary [147,148,149], and lung [52,150,151,152] cancers have highlighted core fucosylation and specific core fucosylated proteins as prognostic serum and tissue biomarkers (Table 6).

Generally, core fucosylation has been reported to be increased in tumor tissues compared to normal tissues, suggesting tumor-promoting functions. Several studies have reported that the silencing of FUT8 in cultured prostate, melanoma, lung, breast, and HCC cancer cells that express high levels of FUT8 inhibits invasion [95,140], migration [140,153], proliferation [52,140], colony formation [52], tumor growth [5,50,95], and metastasis [5,95].

**Figure 4:**
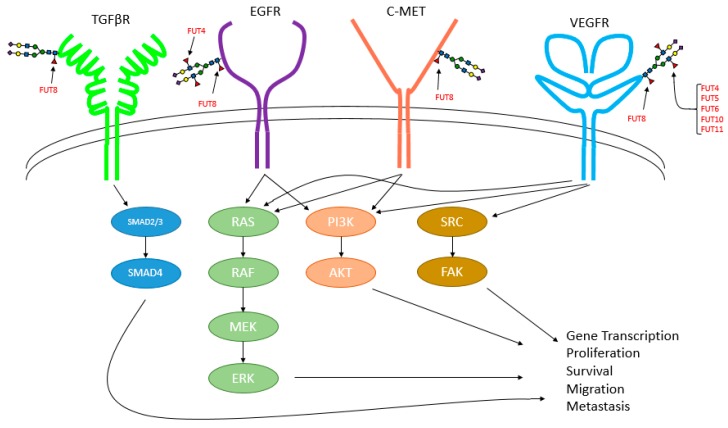
Growth factor signaling pathways known to be regulated by fucosylation.

The specific functional effects elicited by core fucosylation are attributed to its regulation to a number of important growth factor signaling pathways including those mediated by TGFβ [5,158], EGFR [6,50,158,159], VEGFR [158], and c-Met [50] (Figure 4). Fucosylation also impacts the activity/signaling of other plasma membrane proteins including β1-integrin [159], β-catenin [155,160], and E-cadherin [52,160]. Knockdown or inhibition of FUT8/suppression of core fucosylation attenuates these signaling pathways, suppressing cancer growth/survival in vitro models of lung cancer and HCC. [6,50] In addition, FUT8 knockdown or lectin blockade (i.e., incubation of cells with unconjugated LCA lectin, which binds/blocks α(1,6) linkages) inhibits breast cancer stemness and EMT [154].

However, gastrointestinal cancer studies have reported discrepant findings, where three studies have reported reduced core fucosylation in tumors [144,146,161], whereas two reported increased core fucosylation in tumors [89,145]. Interestingly, a number of studies have reported low levels of core fucosylation in gastric cancer cells [146,162], giant lung cancer cells [163], and HCC cells [164] and that the overexpression of FUT8 in those cancer cells suppresses proliferation, tumor formation, and metastasis. The mechanism(s) underlying these differences, as well as the pathological contexts and functional roles remain unclear, in large part because our understanding of how FUT8 is regulated remains limited.

At the transcriptional level, FUT8 has been reported to be transcriptionally activated by p53 [165]. As p53 is often inactivated in cancer, aberrant upregulation of FUT8 might be attributed to the activity of other as-of-yet undefined transcription factors. Post-transcriptionally, FUT8 has been reported to be regulated by miR-122 and miR-34, which bind to the 3′ UTR of FUT8 mRNA transcripts, inhibiting its expression, and consequently, reducing core fucosylation [166]. The roles of these and other miRNAs in the control of FUT expression and fucosylation in cancer remain to be determined. Furthermore, the contributions of these FUT8-regulating mechanisms likely vary by pathological context, resulting in diversity of core fucosylation across cancers.

The divergent regulation of FUT8 and resulting core fucosylation levels can be regulated in stage- and other clinical context-specific manners. The stage-wise importance and contributions of core fucosylation to tumorigenesis has been clearly illustrated in a mouse model of HCC development, where FUT8 activity is required for the development of well-vascularized tumors, whereas knockout of FUT8 completely abolishes tumor formation [50]. In the context of cancer cell responses to therapies, FUT8 has been reported to promote drug resistance. FUT8 expression is increased in drug resistant HCC cells, and its knockdown attenuates Akt-mediated survival signaling [131]. In prostate cancer, FUT8 is upregulated in castration-resistant cells and can mediate the survival and proliferation of non-resistant cells in castrate-(hormone-depleted) conditions [157]. Interestingly, a commonly administered opioid analgesic for cancer patients, fentanyl, was reported to promote breast cancer progression by upregulating FUT8 and enhancing α(1,6) fucosylation, highlighting the unanticipated and confounding effects that therapeutic clinical agents have on fucosylation [154]. These findings prompt the question of whether pain management agents inadvertently promote core fucosylation-mediated cancer progression. How fucosylation and which FUTs are affected by opioids and other supportive agents represents an important and understudied area. Importantly, as FUT8 is the only known fucosyltransferase to mediate core fucosylation, it might prove to be a valuable target for cancer therapy.

## 8. O-Fucosylation

O-fucosylation has been extensively studied for its biological functions in protein folding, secretion [167,168], and in the regulation of Notch signaling [169,170,171,172,173]. Given these biological roles, O-fucosylation is anticipated to impact several functional hallmarks and signaling pathways in cancer that have yet to be defined. Currently, limited data supports this notion. in vitro studies have revealed that aberrant expression of POFUT1 promotes tumorigenic behavior in HCC lines by altering Notch signaling, and in addition, upregulated POFUT1 expression in human HCC tissue specimens correlates with poor overall survival outcomes and increased recurrence rates [174]. In contrast, increased POFUT1 expression in breast cancers is intriguingly associated with longer relapse free and overall survival [175]. The divergent effects of O-fucosylation between different cancer types highlights the need for further studies to elucidate the underlying mechanistic differences.

## 9. Potential Therapeutic Utility

In regard to therapeutic approaches and clinical utility, L-fucose, fucose-containing extracts, inhibitors of fucosylation, and fucosylated liposomes have been investigated as potential therapeutic agents for various cancer types. The administration of L-fucose has been shown to inhibit cell growth in vitro [176] and tumor growth in vivo in breast cancer [177], melanoma [14], lung cancer [4], and Ehrlich carcinoma [178]. As the aberrant expression of certain FUTs appear to elicit tumorigenic effects in tumor cells, it is not immediately clear how the administration of L-fucose inhibits tumor growth and progression. One possible explanation is that the administration of L-fucose increases GDP-fucose substrate availability, boosting the levels of fucosylated glycans with tumor-suppressive properties compared to those with tumorigenic properties. Another possibility is that the administration of L-fucose stimulates anti-tumor immunity [14,179]. Further studies of these phenomena are expected to lead to advances in fucosylation-based therapeutics or dietary interventions for cancer that might slow/block tumor progression or elicit preventative or therapeutic effects.

Currently, L-fucose is relatively expensive and inefficient to purify, which represents a prohibitive factor when considering new treatment options [4]. Furthermore, high levels of L-fucose occur naturally in various species of seaweeds, which can be readily supplemented into current diets. Seaweed-derived L-fucose extracts (known as fucoidan) have been analyzed and shown to elicit anti-tumorigenic responses in breast cancer [180,181,182] and CRC [181,183,184]. Several studies have reported tumor suppressive properties of fucoidan [185,186,187,188,189,190,191,192,193]. Fucoidan treatment is associated with reduced VEGF and Hif1α expression, reduced activation of ERK, inhibited angiogenesis, and attenuated lung cancer cell proliferation, migration, and tumor volume [185]. Fucoidan treatment can also block the angiogenesis-promoting abilities as well as the viability of anaplastic thyroid cancer cells [192]. In lung cancer xenograft models, fucoidan significantly attenuates tumor growth by enhancing ER stress-induced apoptosis. Whereas fucoidan does not affect the proliferation of OSCC cells, it inhibits their invasive capacity, and further, modulates their interactions with macrophages [186]. Treatment of melanoma cells with fucoidan is associated with reduced tyrosinase activity and melanin content, as well as decreased viability [188]. In combination with the ERBB inhibitor lapatinib, fucoidan was reported to enhance melanoma cell killing without adverse effects in mouse models [191]. Fucoidan also elicits dose-dependent effects in prostate cancers cells, reducing cell viability/proliferation, migration, tube formation, tumor volume, and activation of the JAK/STAT pathway. [189] Primary effusion lymphoma cells treated with fucoidan exhibit inhibited proliferation, tumor burden, and enhanced apoptosis as evidenced by increased expression of cleaved capsase-3, -8, -9, and cleaved PARP [190]. Although the use of fucoidan appears beneficial in cancer treatment, further studies, such as those elucidating bioavailability, pharmacokinetics, and pharmacodynamics are required to delineate how and when they should be administered to patients for maximum benefit.

Within the past decade, inhibitors of fucosylation have emerged as potential therapeutic agents under investigation for their ability to inhibit cancer progression. Fluorinated and alkynated fucose analogs have been developed to suppress the synthesis of GDP-fucose, thereby preventing FUTs from fucosylating glycans [111,159,194,195,196]. The treatment of breast cancer cell lines with 2-fluorofucose inhibits sLe^x^ antigen expression, leading to reduced adhesion but not viability [111]. The treatment of HCC cell lines with 2-fluorofucose inhibits core fucosylation, cell proliferation, migration, and tumor formation [159]. Recently, 6-alkyl-fucose was shown to be a more potent than 2-flurofucose in inhibiting fucosylation and viability in HCC cells [196]. Further studies aimed at determining how fucosylation profiles change or are restored in cancer cells after treatment with fucosylation inhibitors or with L-fucose/fucoidan are expected to clarify how they can be used to help suppress or prevent which types of cancer.

Immunotherapies and combination treatments are becoming leading topics in cancer treatment. T cells are one of the cytotoxic populations of the adaptive immune system that require core fucosylation of the T cell receptor to be activated in disease [197,198,199,200]. One of the more extensively investigated immunotherapies is immune checkpoint blockade, specifically antibodies that target and disrupt the interaction between programmed death 1 (PD-1) and its cognate ligand (PD-L1). Interestingly, a defucosylated antibody engineered to disrupt the PD-1/PD-L1 interaction by binding to PD-L1 was recently reported to more effectively induce T cell response and cytotoxicity against cancer cells than fucosylated counterparts [201]. Briefly, PD-L1 on the cell surface of tumor cells interacts with PD-1 on T cells to induce exhaustion, thus impairing the cytotoxic effects of T cells, [202] leading to immune evasion and continued tumor growth. PD-L1 on tumor cells is glycosylated, which contributes to its stabilization at the cell surface [203]. Similar to the glycosylation of PD-L1 in tumor cells, the fucosylation of PD-1 was recently reported to promote its stabilization and presentation at the surface of T cells. Murine T cells inhibited for core fucosylation by Fut8 knockout or pharmacological inhibition (2-fluorofucose) exhibited reduced PD-1 expression and were more cytotoxic and effective at killing melanoma and lung cancer cells [204]. Investigations examining the clinical efficacy of fucosylation inhibitors targeting FUT8 in human T cells will need to be conducted.

## 10. Conclusions and Closing Remarks

Cancer development and tumor progression require pathogenic alterations to normal cellular biology. Increasing research efforts, including those investigating the roles of fucosylation in cancer, are focusing on determining how aberrant glycosylation mechanistically contributes to tumorigenesis and metastatic progression (Table 7).

Although trends in fucosylated glycan structures have been identified among several cancer types (Table 8), many questions remain regarding the differential roles of such types of fucosylation in cancer pathogenesis. Specific fucosylated proteins and the signaling mechanisms that they regulate are just beginning to be elucidated.

Few studies have investigated the functional contributions of cell surface fucosylation during different stages of tumorigenesis, from invasion into local tissues, basement membrane, and the lymphatics and vasculature during metastatic progression [5,87,88,151,159]. Expanding and determining how such mechanistic insights can be used to improve diagnostic or treatment strategies for cancer are expected to improve patient outcomes.

Our understanding of the importance of fucosylation in cancer has undergone significant expansion since studies in the early 1960s. Despite the current complexity of fucosylation and cancer progression, increasing studies are actively elucidating the underlying mechanisms and applications of L-fucose, fucose analogs, and specific aspects of fucosylation to enhance the detection of and therapeutic interventions for multiple cancer types, ultimately aiming to improve clinical outcomes for patients.

## Figures and Tables

**Table 1:** Lectins commonly used in for the detection of specific branches of fucosylation.

Fucosylation	α(1,2)	α(1,3/4)	α(1,6)
Ulex Europaeus Agglutinin 1 (UEA-1)	X		
Aleuria Aurantia Lectin (AAL)		X	X
Lens Culinaris Agglutinin (LCA)			X
Lotus Tetragonolobus Lectin (LTL)	X		

**Table 2:** Fucosylation linkages, associated FUTs, and targets

Structural Linkage	FUTs	Targets & Functions	Refs
α(1,2)	FUT1	H, ABO, and Lewis^y^ (Le^y^) antigen synthesis; endothelial cell tube formation; leukocyte-synovial fibroblast proliferation/adhesion; thymocyte maturation (T cell receptor signaling/apoptosis)	[33,34,35,36]
	FUT2	leftH and ABO antigen synthesis	[37,38,39,40]
α(1,3) α(1,4)	FUT3	Le^a^, Le^b^, Le^x^, and sialyl-Le^x^ (sLe^x^) antigen synthesis	[31,41]
	FUT4/5	Sialylated precursor selectin ligands (leukocyte biology); Le^a^, Le^b^, Le^x^, and sialyl-Le^x^ (sLe^x^) antigen synthesis	[28,30,31,42,43,44]
	FUT6	Le^x^ and sLe^x^ antigen synthesis	[28,31,45]
	FUT7	Sialylated precursor selectin ligand synthesis (leukocyte biology)	[1,31,46]
	FUT9	Le^x^ antigen synthesis	[31]
	FUT10/11	Le^x^ and sLe^x^	[47,48]
α(1,6)	FUT8	TGFβR; EGFR; METR; E-cadherin, T-cell receptor	[3,6,11,27,31,49,50,51,52]
O-fucosylation	POFUT1/2	Epidermal Growth Factor-like and Thrombospondin Type 1 repeats of proteins	[19,20,21]

**Table 3:** Altered serum fucosylation profiles in cancer patients.

Cancer Type	Changes in Serum Fucosylation	Refs
Breast	• Increased free L-fucose	[53,54]
	• Increased serum FUT activity	[76]
	• Increased fucosylated haptoglobin	[77]
	• α(1,3) fucosylation is increased in cancer patients	[54,55]
Oral/Head & Neck	• Increased free L-fucose	[56,57,58,59,60,61]
Liver	• Increased free L-fucose	[62,63]
	• Increased fucosylated haptoglobin	[69,70,71,78]
	• Core fucosylation of haptoglobin is increased in cancer patients	[69]
	• Increased fucosylated α-fetoglobin in serum of cancer patients	[3,31,75]
Ovarian	• Increased free L-fucose	[68]
	• FUT3 found to be circulating in serum	[55]
	• Increased levels of fucosylated proteins in cancer patients	[79]
	• Increased fucosylated haptoglobin	[31,55,72]
Prostate	• Increased levels of fucosylated proteins in cancer patients	[80]
	• PSA from patient serum is α(1,2) fucosylated	[81]
	• PSA from patient serum is α(1,6) fucosylated	[82]
	• Core fucosylation of PSA in urine decreases as disease progresses	[83]
Colorectal	• Increased free L-fucose	[66]
	• Increased serum FUT activity	[4]
	• Increased fucosylated haptoglobin	[67]
	• α(1,3) fucosylation is increased in cancer patients	[67]
Pancreatic	• Increased fucosylated haptoglobin	[73]
Lung	• Smoking increases the level of fucosylated proteins in cancer patients	[84]
	• Increased fucosylated haptoglobin	[31,74]
	• Increased core fucosylation of serum E-cadherin in cancer patients	[51]

**Table 4:** Alterations and roles of α(1,2) fucosylation in tumors.

Cancer Type	Alterations and Roles of α(1,2) Fucosylation	Refs
Melanoma	• α(1,2) fucosylation inhibits tumor formation	[14,15,87]
	• 25% of melanoma cell lines lack FUT1 expression	[11,94]
	• FUT1 expression is decreased in tumors	[14,95]
	• α(1,2) fucosylation inhibits invadopodia & invasion	[87]
Oral/Head & Neck	• α(1,2) fucosylation inhibits tumor formation	
	• FUT1 expression is decreased in tumors	[16]
	• α(1,2) fucosylation high in tumors, lost at invading front	
Gastric	• α(1,2) fucosylation inhibits tumor formation	[89]
Hepatocellular	• α(1,2) fucosylation inhibits tumor formation	[17,63]
	• FUT1 expression is decreased in tumors	[63]
Ovarian	• α(1,2) fucosylation is increased by FUT1 upregulation	[96]
Prostate	• α(1,2) fucosylation is increased by FUT1 upregulation	[81,85]
Colorectal	• α(1,2) fucosylation increased in tumor tissues	[15,97,98,99]
	• FUT1 expression attenuates adhesion and metastasis to the liver	
Pancreatic	• α(1,2) fucosylation is decreased in primary tumor tissues.	[86,100,101,102]
	• FUT1 expression decreases metastatic adhesion	[86]
Breast	• FUT1 mRNA is upregulated in adriamycin-resistant cells	[92]
	• α(1,2) fucosylation regulates autophagic flux	[91]
Bladder	• α(1,2) fucosylation promotes cell adhesion	[93]
Epidermoid	• α(1,2) fucosylation promotes cell proliferation	[90]

**Table 5:** Alterations of α(1,3/4) fucosylation tumors.

Cancer Type	Changes in α(1,3/4) Fucosylation	Reference
Breast	• α(1,3/4) fucosylation upregulated in tumor tissue	[110,111,112]
Melanoma		[124]
Oral/Head & Neck		[125]
Liver		[63,113]
Ovarian		[114,115]
Prostate		[121,122,123]
Colorectal		[97]
Pancreatic		[100,116,117]
Gastric		[118,119]
Lung		[120]

**Table 6:** Alterations of core fucosylation in tumors.

Cancer Type	Changes in α(1,6) Fucosylation	Reference
Breast	• Core fucosylation increased in tumor tissue	[139,154]
Melanoma		[95]
Liver		[11,63,140,155]
Ovarian		[11,68,141,156]
Cervical		[141]
Colorectal		[11,97,142,143]
Pancreatic		[117]
Lung		[52,150,151,152]
Gastric	• Core fucosylation increased in tumor tissue	[89,145]
	• Core fucosylation decreased in tumor tissue	[144,146]
Prostate	• Core fucosylation increased in castrate resistant tissue	[157]

**Table 7:** Summary of studies that have manipulated FUTs and documented biological outcomes in tumor cell lines.

Cancer Type	Results of FUT Manipulation in Cell Lines	Reference
Breast	• FUT4 overexpression promoted invasion & tumor growth	[130]
	• FUT8 knockdown inhibited tumor growth & metastasis	[5]
	• FUT8 overexpression promoted EMT and invasion	[5]
Melanoma	• FUT1 overexpression inhibited metastasis	[15]
	• FUT1 overexpression inhibited invadopodia & invasion	[87]
	• FUT4 knockdown inhibited proliferation & tumor growth	[129]
	• FUT8 knockdown decreased invasion, tumor growth, & metastasis	[95]
Oral/Head & Neck	• FUT1 overexpression suppressed cell growth & invasion; knockdown increased cell growth &invasion	[16]
	• FUT3 overexpression promoted invasion	[125]
	• FUT6 overexpression enhanced adhesion & invasion	[125]
Liver	• FUT1 overexpression suppressed adhesion	[17]
	• FUT6 overexpression increased proliferation, colony formation, & tumor growth	[113]
	• FUT4, 6, & 8 overexpression amplified drug resistance	[131]
	• FUT4, 6, & 8 knockdown suppressed drug resistance and inhibited tumor growth	[131]
	• FUT7 silencing decreased adhesion, migration, & invasion	[134]
	• FUT7 overexpression amplified proliferation	[138]
	• FUT8 knockdown inhibited invasion, migration, & proliferation	[140]
	• FUT8 overexpression suppressed proliferation, tumor formation, & metastasis	[164]
Ovarian	• FUT1 overexpression increased proliferation adhesion, invasion, metastasis & resistance	[108,205]
	• FUT1 overexpression increased colony formation & proliferation	[103]
Prostate	• FUT3 overexpression amplified adhesion	[133]
	• FUT6 overexpression increased migration & metastasis	[133]
	• FUT7 overexpression enhanced adhesion	[133]
	• FUT8 knockdown decreased migration	[153]
	• FUT8 overexpression increased motility	[153]
Colorectal	• FUT1 overexpression suppressed adhesion	[97]
	• FUT1 overexpression inhibited metastasis	[109]
	• FUT3 & 6 knockdown decreased adhesion, invasion, & migration	[127]
	• FUT5/6 knockdown inhibited migration and proliferation	[206]
Pancreatic	• FUT1 overexpression suppressed adhesion and metastasis	[86]
	• FUT3 knockdown decreased migration, adhesion, and metastatic colonization	[116,207]
Gastric	• FUT3 knockdown decreases migration	[119]
	• FUT5 knockdown inhibited adhesion & migration	[119]
	• FUT8 overexpression suppressed proliferation, tumor formation, & metastasis	[146]
Lung	• FUT4 overexpression promoted EMT	[208]
	• FUT7 overexpression increased adhesion, colony formation, invasion, & migration	[137]
	• FUT8 knockdown decreased proliferation & colony formation	[52]
	• FUT8 overexpression suppressed proliferation, tumor formation, & metastasis	[163]

**Table 8:** Visual summary of fucosylation changes of the branching types in cancer tissues vs. normal tissues. ↑-increased; ↓-decreased; ↑→↓-increased in primary, decreased in metastasis.

Cancer Type	α(1,2)	α(1,3/4)	α(1,6)
Breast	-	↑	↑
Melanoma	↓	↑	↑
Oral/Head & Neck	↓	↑	-
Liver	↓	↑	↑
Ovarian	↑	↑	↑
Prostate	↑	↑	↑
Colorectal	↓/↑	↑	↑
Pancreatic	↑ → ↓	↑	↑
Gastric	-	↑	↓/↑
Lung	-	↑	↓/↑

## Abbreviations

CRC	colorectal carcinoma
EGF	epidermal growth factor
ELISA	enzyme-linked immunosorbent assay
EMT	epithelial-mesenchymal transition
FGF	fibroblast growth factor
FUK	fucokinase
FUT	fucosyltransferase
FX	NADP(H)-binding epimerase-reductase
GlcNAc	N’-acetylglucosamine
GMD	GDP-mannose 4,6-dehydratase
HPLC	high performance liquid chromatography
HCC	hepatocellular carcinoma
LAMP1	lysosome-associated membrane protein 1
LC	liquid chromatography
MALDI	matrix assisted laser desorption/ionization
MS	mass spectrometry
NSCLC	non-small cell lung cancer
OSCC	oral squamous cell carcinoma
POFUT	protein-O-FUT
sLe	sialyl Lewis
TGFβ	tumor growth factor beta
THBS	thrombospondin
VEGF	vascular endothelial growth factor
